# Cell Loss and Autophagy in the Extra-Adrenal Chromaffin Organ of Zuckerkandl are Regulated by Glucocorticoid Signalling

**DOI:** 10.1111/j.1365-2826.2012.02367.x

**Published:** 2012-12-21

**Authors:** Andreas Schober, Rosanna Parlato, Katrin Huber, Ralf Kinscherf, Björn Hartleben, Tobias B Huber, Günther Schütz, Klaus Unsicker

**Affiliations:** * Department of Molecular Embryology, Institute of Anatomy and Cell Biology II, Albert-Ludwigs-University FreiburgFreiburg, Germany; † Division of Molecular Biology of the Cell I, DKFZ-ZMBH Alliance, German Cancer Research CenterHeidelberg, Germany; ‡ Anatomy and Cell Biology, Department of Medical Cell Biology, Philipps-University of MarburgMarburg, Germany; § Renal Division, University Hospital FreiburgFreiburg, Germany; # BIOSS Centre for Biological Signalling Studies, Albert-Ludwigs-University FreiburgGermany

**Keywords:** glucocorticoid receptor, conditional deletion, neuroendocrine chromaffin cells, autophagic cell death, Atg5 deficient mice

## Abstract

Neuroendocrine chromaffin cells exist in both intra- and extra-adrenal locations; the organ of Zuckerkandl (OZ) constitutes the largest accumulation of extra-adrenal chromaffin tissue in mammals. The OZ disappears postnatally by modes that are still enigmatic but can be maintained by treatment with glucocorticoids (GC). Whether the response to GC reflects a pharmacological or a physiological role of GC has not been clarified. Using mice with a conditional deletion of the GC-receptor (GR) gene restricted to cells expressing the dopamine β-hydroxylase (DBH) gene [GR^fl/fl^; DBHCre abbreviated (GR^DBHCre^)], we now present the first evidence for a physiological role of GC signalling in the postnatal maintenance of the OZ: postnatal losses of OZ chromaffin cells in GR^DBHCre^ mice are doubled compared to wild-type littermates. We find that postnatal cell loss in the OZ starts at birth and is accompanied by autophagy. Electron microscopy reveals autophagic vacuoles and autophagolysosomes in chromaffin cells. Autophagy in OZ extra-adrenal chromaffin cells is confirmed by showing accumulation of p62 protein, which occurs, when autophagy is blocked by deleting the Atg5 gene (*Atg5*^*DBHCre*^ mice). Cathepsin-D, a lysosomal marker, is expressed in cells that surround chromaffin cells and are positive for the macrophage marker BM8. Macrophages are relatively more abundant in mice lacking the GR, indicating more robust elimination of degenerating chromaffin cells in GR^DBHCre^ mice than in wild-type littermates. In summary, our results indicate that extra-adrenal chromaffin cells in the OZ show signs of autophagy, which accompany their postnatal numerical decline, a process that is controlled by GR signalling.

Chromaffin cells are neuroendocrine cells derived from the neural crest 1,2. They populate the adrenal medulla and extra-adrenal sympathetic paraganglia. The largest accumulation of extra-adrenal chromaffin cells is the organ of Zuckerkandl (OZ) 3–6.

The physiological role of the OZ is not clear, although it has been suggested to relate to homeostatic regulation of blood pressure during early gestation, secreting catecholamines into the foetal circulation 7. Other para-aortic assemblies of chromaffin cells serve as chemoreceptors responsive to oxygen, carbon dioxide and hydrogen ion concentration, and support the control of respiration 8–11.

The OZ regresses after birth according to a species-specific schedule; in humans, it reaches its maximal size at the age of approximately 3 years, and, in mice, around birth 4,12,13. The mechanisms by which the chromaffin cells in the OZ disappear postnatally are largely obscure and may involve loss of phenotype, ‘transdifferentiation’ into sympathetic neurones, dispersion by migration and/or degeneration/cell death 7,14,15. Extra-adrenal (similar to intra-adrenal) chromaffin cells are of pathological significance in the adult as a common site of the origin of pheochromocytoma. Extra-adrenal tumors account for approximately 10% of all cases of pheochromocytoma 16,17. Furthermore, the OZ has attracted interest as a source of chromaffin tissue for grafts to the striatum in animal models of Parkinson's disease 18,19.

The close spatial association of adrenal medullary chromaffin cells and steroid hormone producing cells of the adrenal cortex has always fostered speculation concerning a role of glucocorticoids (GC) in the determination and differentiation of the chromaffin cell phenotype. In support of this notion, GC are essential for the induction of the adrenaline synthesising enzyme phenylethanolamine N-methyltransferase (PNMT) in a subpopulation of mammalian adrenal chromaffin cells 20. Suppression of the neuronal differentiation in isolated embryonic and early postnatal cells cultured from sympathetic ganglia and adrenal medulla by GC 21,22 generated the hypothesis that GC signalling might be important for the specification of the chromaffin as opposed to the neuronal phenotype. However, the hypothesis was not validated *in vivo* by the phenotype of mice lacking a functional glucocorticoid receptor (GR) 23. GR knockout mice, which die at birth as a result of lung failure, have normal numbers of adrenal chromaffin cells but show some alterations in a restricted set of molecular markers, including PNMT. However, conditional inactivation of the GR gene induces progressive apoptotic cell death of chromaffin cells in the adrenal medulla after birth, suggesting a requirement of GC for the postnatal maintenance of adrenal chromaffin cells 24. With regard to extra-adrenal chromaffin tissues, numerous studies have shown that application of GC causes hyperplasia of chromaffin cells 3,25–29.

The present study investigates the mode and regulation of the physiological regression of the OZ. Mice with a conditional deletion of the GR gene under the control of the dopamine ß-hydroxylase (DBH) promoter (*GR*^*DBHCre*^) are viable and allow postnatal analysis of the OZ. We show that loss of GR signalling significantly accelerates the disappearance of chromaffin cells in the OZ. This suggests that GR signalling is physiologically relevant for the postnatal maintenance of the OZ and that a postnatal decline in GR signalling may account for the physiological disappearance of the OZ. The decline of chromaffin cells in the OZ is accompanied by the morphological signs of autophagy but not of apoptosis.

## Materials and methods

### Animals

The following total numbers of animals at embryonic (E) and postnatal ages (P) of C57Bl6 wild-type and *GR*^*DBHCre*^ mice were used in the present study: wild-type, E16 (n = 3), E18 (n = 6), P1 (n = 4), P3 (n = 16), P6 (n = 16), P10 (n = 10); *GR*^*DBHCre*^, E18 (n = 3), P1 (n = 6), P3 (n = 10), P6 (n = 10), P10 (n = 6). Wild-type and *GR*^*DBHCre*^ mice were generated in the laboratory of G. Schütz (German Cancer Research Center, Heidelberg, Germany). In addition, for the immunohistochemical detection of autophagy in extra-adrenal chromaffin cells of OZ, Atg5 mutant mice (*Atg5*^*DBHCre*^ and corresponding wild-type mice) were used (P6). Atg5 mutant mice (*Atg5*^*flox/flox*^) were generated in the laboratory of Noboru Mizushima (Tokyo, Japan) and crossed to *DBHCre* mice 30. Genotypings were carried out by polymerase chain reaction (PCR) analysis as described previously 24,30. All animal experiments were approved by the local animal care committee.

### Tissue preparation

Pregnant mice were killed by CO_2_ asphyxiation. Embryos were recovered, rinsed in cold phosphate-buffered saline (PBS) (pH 7.4) and fixed in PBS containing 4% paraformaldehyde (PFA) overnight. Postnatal mice of the age P1, P3, P6 and P10 were anaesthetised and transcardially perfused with 4% PFA as described previously 31. For all stages investigated, a thick abdominal body segment (approximately 1–1.5 cm), containing the area of the renal pelvis, including adrenal glands, kidneys and the OZ, was removed and immersed in the same fixative. Segments were then placed in 30% sucrose for cryoprotection and, finally, frozen on dry ice by coating with OCT compound (Tissue Tek; Sakura Finetek USA, Inc., Torrance, CA, USA). Frozen samples were stored at −20 °C until further processing or subsequently cut into 20 μm serial sections on a cryostat (Leica, Wetzlar, Germany), mounted on Superfrost slides (Fisher Scientific, Hampton, NH, USA), and air-dried for 30 min, before performing *in situ* hybridisation (ISH) or immunofluorescence staining, respectively.

### ISH

Nonradioactive ISH on cryosections and preparation of digoxigenin-labelled probes for mouse neurofilament 68 (NF 68) was carried out using a modification of the protocol of D. Henrique (IRFDBU, Oxford, UK) as described previously 32,33. Mouse NF 68 (gene bank accession number: NM_010910, bp: 418–1112) was cloned by PCR using a pGEM-T vector system in accordance with the manufacturer's instructions. The plasmid was linearised with *Sac*II (antisense) and *Sac*I (sense control) and transcribed with Sp6 (antisense) and T7 (sense control). If required for double-labellings, NF 68-ISH and tyrosine hydroxylase (TH) immunostaining was carried out in combination.

### Immunohistochemistry (IHC)

IHC stainings were performed on 20-μm thick serial cryosections in accordance with established standard protocols 31 and the following primary antibodies (AB) were applied: sheep anti-TH polyclonal (pc) AB (TH, #1542, dilution 1 : 200, in the presence of 0.05% Triton X-100; Chemicon, Temecula, CA, USA), the rat anti-mouse monoclonal (mc) AB to BM8 (F4/80, dilution 1 : 500; BMA Biomedicals, Augst, Switzerland) used as a pan macrophage marker, the pcAB to cleaved caspase-3 (dilution 1 : 200, # 9661; Cell Signaling Inc., Beverly, MA, USA) and the pcAB to mouse cathepsin-D (dilution 1 : 2000, kindly provided by K. von Figura, Göttingen, Germany) 34 used as a lysosomal marker. For monitoring autophagy, a rabbit pcAB to Atg5 (dilution 1 : 100, AP1812a; Abgent Inc., San Diego, CA, USA) and a guinea pig pcAB to p62 protein (dilution 1 : 200; Progen, Heidelberg, Germany, GP62-C, visualised by Alexa Fluor 488 anti-guinea pig immunoglobulin (Ig)G, 1 : 500, 30 min, room temperature) were used.

Double-labellings were performed by adding two primary antibodies successively to the same section: first BM8 (dilution 1 : 500; in the presence of 0.05% Triton X-100, overnight, room temperature) and second TH (dilution 1 : 200, in the presence of 0.05% Triton X-100 overnight, room temperature). Sections incubated with mcAB BM8 were subsequently treated with biotinylated anti-rat IgG (dilution 1 : 100, 2 h, room temperature; GE Healthcare Europe, Freiburg, Germany); and then visualised by Cy3-conjugated streptavidin (dilution 1 : 200, 2 h; room temperature; Jackson ImmunoRes by DIANOVA, Hamburg, Germany). Thereafter, the same sections were incubated with pcAB to TH as described before and visualised by Cy2-conjugated anti-sheep IgG (dilution 1 : 200, 2 h, room temperature; Jackson ImmunoRes by DIANOVA).

Among the several incubation steps, sections were several times rinsed in PBS. Nuclei were counterstained with 4′,6-diamidino-2-phenylindole (dilution 1 : 10 000, 2 min, room temperature; Sigma, Munich, Germany). Finally, all sections were rinsed three times in PBS and embedded in fluorescent mounting medium (DAKO, Glostrup, Germany).

### Antibody characterisation

**Fig. 1:**
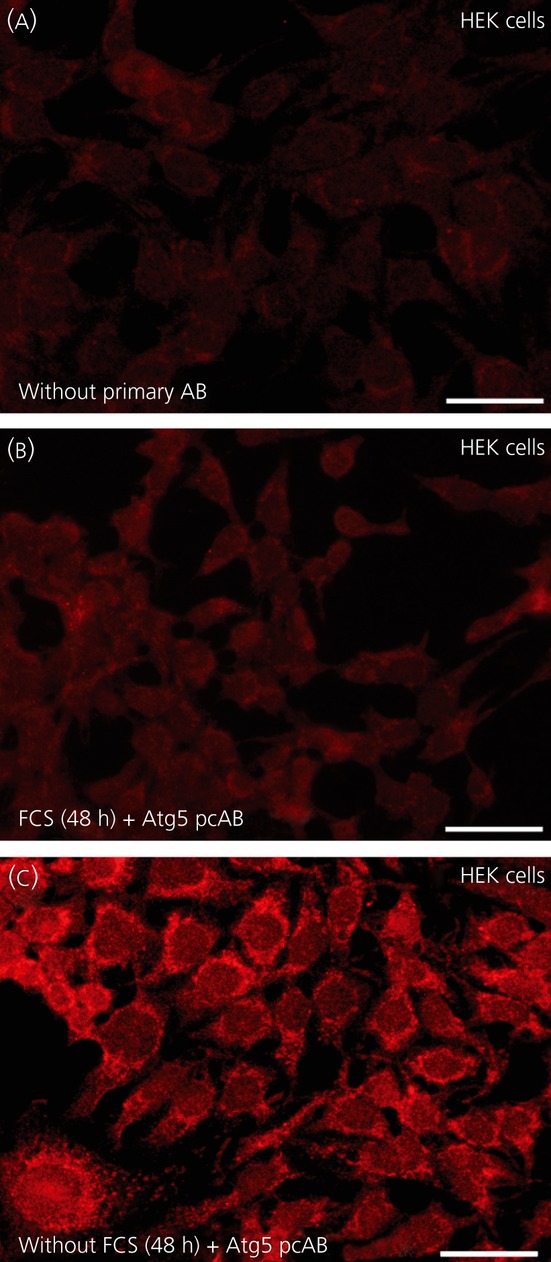
The specificity of the applied pcAB to Atg5 as a specific autophagy marker has been tested *in vitro* on starved and foetal calf serum (FCS)-treated human embryonal kidney (HEK) cells. (a) Negative control without primary antibody. (b) HEK cells were treated for 48 h with FCS, autophagy induction is not detectable by Atg5-immunoreactivity (-IR). (c) Showing the induction of autophagy in starved HEK cells by a specific pattern of Atg5-IR. Unfortunately, the same Atg5 pcAB did not stain autophagic cells in paraformaldehyde-perfused cryosections of mouse tissue. Scale bars = 20 μm.

The optimal antibody concentrations were individually determined for each antibody. Control experiments omitting primary antibodies confirmed always the absence of immunostaining in these conditions. The specificity of the Atg5 antibody was tested *in vitro* on starved and foetal calf serum (FCS)-treated human embryonal kidney (HEK) cells cultured for 48 h. Fig. 1(c) shows the induction of autophagy in starved HEK cells. However, the antibody did not stain autophagic cells in PFA-perfused cryosections of mouse tissues.

#### TH pcAB

For TH staining, an affinity-purified sheep anti-TH pc antibody (Chemicon, catalogue number AB1542, dilution 1 : 200) has been used. This antibody was raised against a sodium dodecyl sulphatedenaturated TH from rat pheochromocytoma (Chemicon datasheet). It recognises a single band of 62 kDa molecular weight on western blotting from PC12 cells stimulated with okadaic acid (Chemicon datasheet). The staining obtained with this antibody is cytoplasmic and is present in both cell bodies and fibres. The specificity of this antibody is supported by the location of the staining (e.g. we observed TH-positive cell bodies and fibres in the sympathetic ganglia, in chromaffin cells of the adrenal medulla and in extra-adrenal chromaffin cells in the OZ).

#### BM8 mcAB

The applied affinity purified mc antibody BM8 recognises the F4/80 antigen (dilution 1 : 500, catalog T-2006, lot 13PO0804, clone: BM8; BMA Biomedicals) on major subpopulations of resident tissue macrophages. The BM8 antibody was raised against a 125-kDa extracellular membrane protein (sensitive to 2-mercaptoethanol), isolated from cultured macrophages.The antigen is expressed *in vitro* on over 80% of macrophage colony-stimulating factor stimulated bone marrow derived macrophages after a few days of culture. It is absent from granulocytes, lymphocytes and thrombocytes (catalogue T-2006; BMA Biomedicals).

#### Caspase-3 pcAB

Rabbit cleaved caspase-3 (Asp175) pc antibody (dilution 1 : 200, catalogue 9661; Cell Signaling Inc.), which was raised against a synthetic peptide (KLH coupled) corresponding to amino-terminal residues (CRGTELDCGIETD) adjacent to (Asp175) in human caspase-3. This antibody detects endogenous levels of the large fragment (17/19 kDa) of activated caspase-3 resulting from cleavage adjacent to Asp175 but does not recognise full-length caspase-3 or other cleaved caspases. Antibodies are purified by protein A and peptide affinity chromatography (catalogue 9661; Cell Signaling). The specific immunostaining is abolished by preincubating the antibody with the cleaved caspase-3 (Asp175) blocking peptide (datasheet, catalogue 1050; Cell Signaling). This antibody is a well described and extensively used marker for apoptosis in mammalian and bird tissues (datasheet, catalogue 1050; Cell Signaling).

#### Cathepsin-D pcAB

For cathepsin-D staining, an affinity-purified mouse anti-cathepsin-D polyclonal antiserum (kindly provided by Klaus von Figura, Göttingen, Germany) 34 has been used. This antibody was raised against cathepsin-D purified from mouse liver cells. The cytoplasmic staining pattern of the antiserum is consistent with the property of labelling lysosomes. The specificity of this antibody is supported by immunoprecipitation comparing wild-type and cathepsin-D knockout tissues.

#### p62

For p62 immunostaining, a stabilised polyclonal antiserum (guinea pig, Progen datasheet, catalogue GP62-C, dilution 1 : 200) was applied. This antibody has been described and extensively used 35 as a marker of autophagy in animal models, in which autophagy has been ablated [e.g. in Atg5 (−/−) mice]. The p62 protein is present in intracytoplasmatic inclusions, also described as the ubiquitin binding protein ‘sequestosome 1’ (SQSTM1; Table 1).

**Table 1:** Primary Antibodies

Antigen	Immunogen	Manufacturer	Dilution
Tyrosine hydroxylase	Rat pheochromocytoma	Chemicon (Temecula, CA, USA; #1542), sheep, polyclonal	1 : 200
Caspase 3	Synthetic peptide, corresponding to amino-terminal residues (CRGTELDCGIETD) in human caspase-3	Cell Signaling(Beverly, MA, USA; # 9661), rabbit, polyclonal	1 : 200
BM8	125 kDa extracellular membrane protein (F4/80) isolated from cultured macrophages	BMA Biomedicals (Augst, Switzerland; T-2006), mouse, monoclonal	1 : 500
Cathepsin-D	Purified cathepsin-D isolated from mouse liver cells	Gift from Dr von Figura (Göttingen, Germany), rabbit, polyclonal	1 : 2000
Atg5	KLH-konjugated synthetic peptide (amino acids 1–30) from N-terminal of human APG5L	Abgent Inc. (San Diego, CA, USA; Ap1812a) rabbit, polyclonal, cross-reactivity: mouse, human	1 : 100
p62	KLH-konjugated C-terminal domain (20 amino acids) of human p62 protein	Progen Biotechnik GmbH (Heidelberg, Germany; GP62-C), guinea pig, polyclonal cross-reactivity: human, mouse, rat	1 : 200

### Electron microscopy (EM)

For standard EM, postnatal mice at P3 and P6 (GR^DBHCre^ and wild-type littermates, n = 6, each) were anaesthetised and perfused using a mixture of glutaraldehyde (1.5%) and PFA (1.5%). After 48 h of postfixation in the same solution, a tissue sample at the level of the renal pelvis was extracted and the adjacent renal and intestinal tissue was removed. Finally, tissue blocks (size: approximately 4 × 4 mm) containing a part of the abdominal aorta, the prevertebral ganglionic complex, consisting of the celiac and the superior mesenteric ganglion and the OZ, were processed for EM as described previously 36. Semithin sections (0.5 μm thick) were taken at 25-μm intervals, transferred onto glass slides, stained with 0.1% toluidine blue in 1% sodium borate and analysed under the light microscope.

### Cell quantification

Determination of absolute chromaffin cell numbers in the OZ of pre- and postnatal knockout and wild-type mice (E16, E18, P1, P3, P6, P10) was performed in 20-μm thick cryosections by profile based counts with Abercrombie-correction for split cells 37,38. This method of quantification was chosen because of the necessity for double-labelling cells with two antibodies, or with an elaborately optimised combination protocol of antibody labelling and ISH. The probes and antibodies did not penetrate tissue sections sufficiently (< 15 μm) to allow use of thick sections, as required for optical disector methodology 39. Only TH-immunoractive (-IR) positive and NF-68 ISH negative cells with a clearly visible nucleus were counted. Every third section through the whole OZ was analysed and extrapolated to estimate the total chromaffin cell number of the OZ of each individual animal. Cryosections double-stained with TH-AB and BM8-AB (P6) were analysed according to the same procedure. All counts were performed with an Axioplan 2 imaging microscope (Zeiss, Göttingen, Germany) using a × 20 objective (NA = 0.5).

Cells showing signs of degeneration in Toluidin blue stained semithin sections (0.5 μm) were determined in a semi-quantitative fashion and such data were analysed in 25-μm intervals (always n = 10) of P3 and P6 animals (wild-type and *GR*^*DBHCre*^). The relative number of chromaffin cells in relation to chromaffin cells showing signs of degeneration was calculated (%).

The mean ± SD were calculated using Origin, version 6.1 (OriginLab Corp., Northampton, MA, USA). The two-tailed t-test was used for statistical evaluation. P < 0.05 was considered statistically significant.

## Results

### Localisation of OZ

**Fig. 2:**
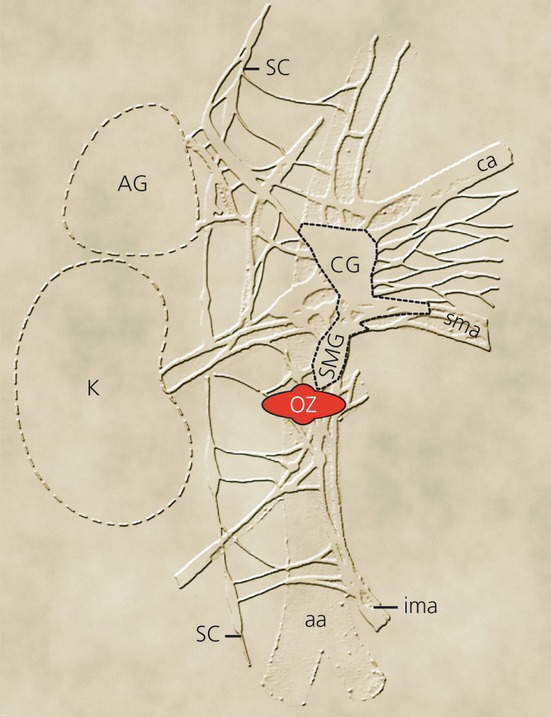
Topographical localisation of the organ of Zuckerkandl (OZ) in newborn mice. aa, abdominal aorta; AG, adrenal gland; ca, coeliac artery; CG+SMG, coeliac + superior mesenteric ganglion complex; K, kidney; ima, inferior mesenteric artery; SC, sympathetic chain; sma, superior mesenteric artery. Modified in accordance with Hamer and Santer 75.

**Fig. 3:**
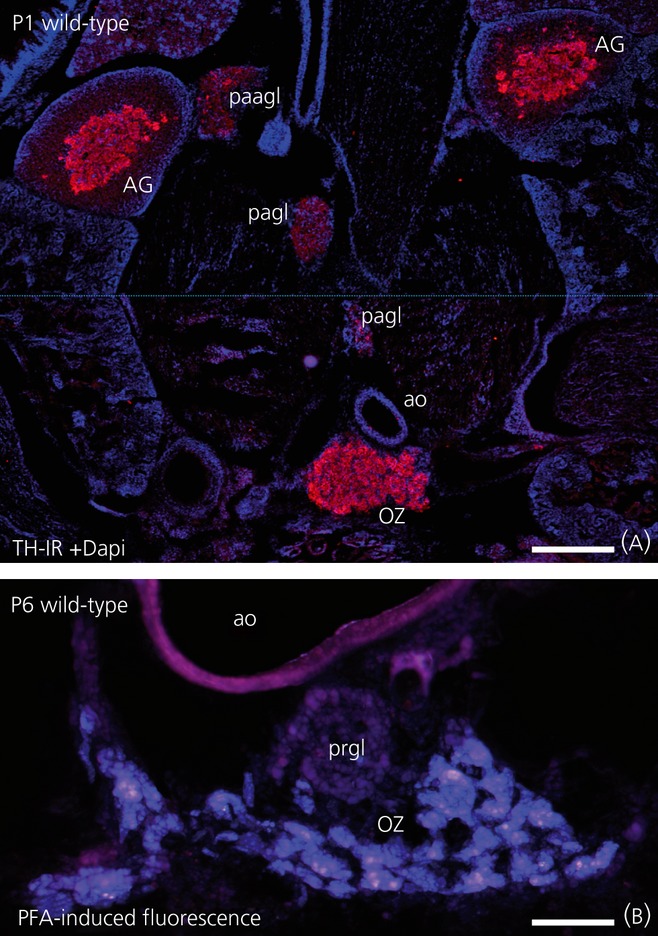
Histological visualisation of the organ of Zuckerkandl (OZ) revealed by tyrosine hydroxylase (TH) -immunoreactivity (-IR) and paraformaldehyde (PFA)-induced histofluorescence. (a) Frontal section through the abdominal region of a P1 wild-type mouse provides an overview of the topography of the OZ in relation to para-aortic sympathetic ganglia (pagl), adrenal gland (AG), and sympathetic para-adrenal ganglia (paagl). Adrenal medullary and extra-adrenal chromaffin cells (OZ) show a very prominent TH-immunofluorescence signal, compared to the weaker staining intensity of sympathetic ganglion cells. Scale bar = 500 μm. (b) PFA-induced histofluorescence can serve as an independent marker for the identification of chromaffin cells, which consistently show stronger histofluorescence than sympathetic neurones. Scale bar = 100 μm. ao, aorta.

**Fig. 4:**
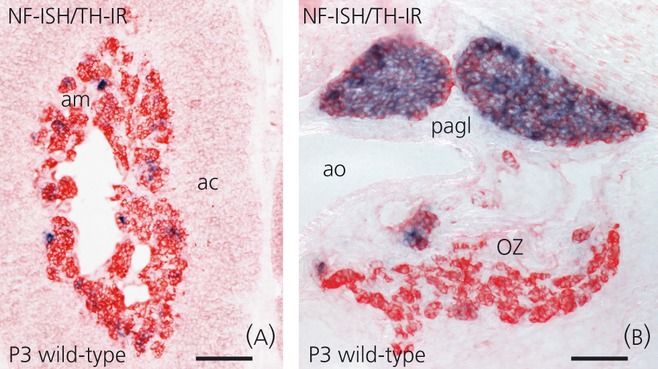
Double-labelling for NF68-*in situ* hybridisation (blue) and tyrosine hydroxylase (TH) -immunoreactivity (-IR) (red) in a P3 wild-type mouse. (a) Chromaffin cells located in the adrenal medulla (am; adrenal cortex, ac) reveal a prominent TH-IR (red), whereas only very few cells express NF68 mRNA (blue). (b) Located dorsally to the abdominal aorta (ao), two paravertebral sympathetic ganglia (pagl) show intense signals for NF68 mRNA and TH-IR, whereas only very few cells in the periphery of the organ of Zuckerkandl (OZ) express NF68 mRNA. Scale bars = 100 μm.

Emil Zuckerkandl, who discovered the OZ (‘Nebenorgane des Sympathicus’ in his terminology), described its localisation in humans as being attached to the abdominal aorta at the level of the origin of the inferior mesenteric artery 6. Today, the whole collection of paraganglia located anterolaterally to the distal abdominal aorta between the origin of the inferior mesenteric artery and the aortic bifurcation is called ‘the organ of Zuckerkandl’ 40. For rodents, two different locations have been published: (i) at the level of the renal vein and renal pelvis 12,13 and (ii) on the abdominal aorta between the origin of the inferior mesenteric and the iliac arteries 18. Because the OZ is localised within a complex of the coeliac and superior mesenteric sympathetic ganglia (Fig. 2), its precise localisation requires methods to unequivocally distinguish between neuroendocrine chromaffin cells and sympathetic neurones. Chromaffin cells express robustly TH, although only very low levels of NF68, whereas sympathetic neurones express TH at lower levels and NF68 at high levels. We therefore employed double ISH/IHC for NF68 and TH, respectively, to differentially mark the two cell types (Figs 3a and 4a,b). Fig. 3(a) reveals prominent TH-IR in chromaffin cells within the adrenal medulla (Figs 3a and 4a) and the OZ (Fig. 4b). Although only very few cells expressing NF mRNA are scattered over the adrenal medulla (Fig. 3a), two paravertebral sympathetic ganglia show intense signals for NF mRNA and TH-IR (Fig. 4b). Figure 3(a) provides an overview of the topography of the OZ in relation to para-aortic sympathetic ganglia, adrenal gland, and sympathetic para-adrenal ganglia. Formaldehyde-induced histofluorescence is an established method for the demonstration of biogenic amines 25,26,41 and can serve as an independent marker for the identification of chromaffin cells, which show stronger histofluorescence than sympathetic neurones (Fig. 3b). Thus, by using combined NF-ISH, TH-IHC and amine histofluorescence, we were able to identify in the mouse the OZ on the anterior surface of the aorta at the level of the renal pelvis (Fig. 2) (i.e. different from its location in man and many rodents) 4.

### Developmental increase and subsequent postnatal decrease of chromaffin cell numbers in the OZ

**Fig. 5:**
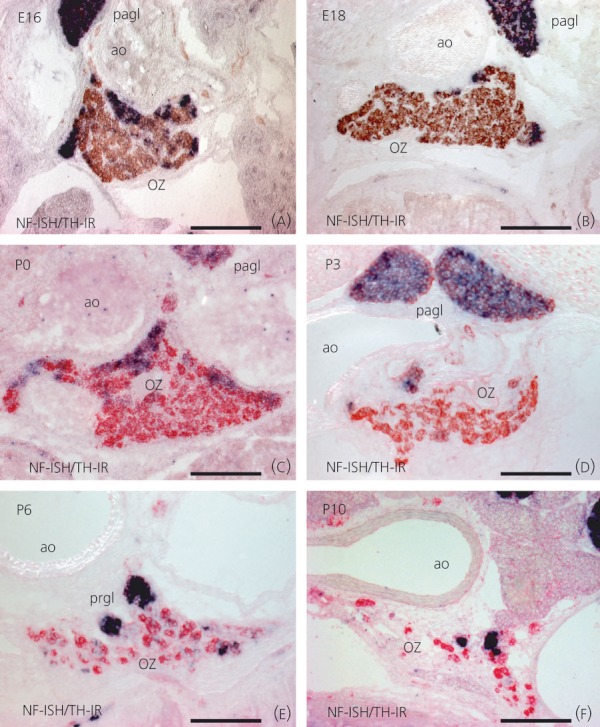
Developmental increase and involution of the organ of Zuckerkandl (OZ) from embryonic day (E) 16 to postnatal day (P) 10. The OZ is always shown at its largest extension. Chromaffin cells and sympathetic neurones can be identified by NF68-*in situ* hybridisation (blue) and tyrosine hydroxylase (TH)-immunoreactivity (-IR) (red) labelling. (a–f) Note that numbers of TH-IR chromaffin cells (red) in the OZ increase until birth and decrease subsequently. Scale bars = 200 μm. ao, aorta; pagl, paravertebral sympathetic ganglia; prgl, prevertebral sympathetic ganglion.

**Fig. 6:**
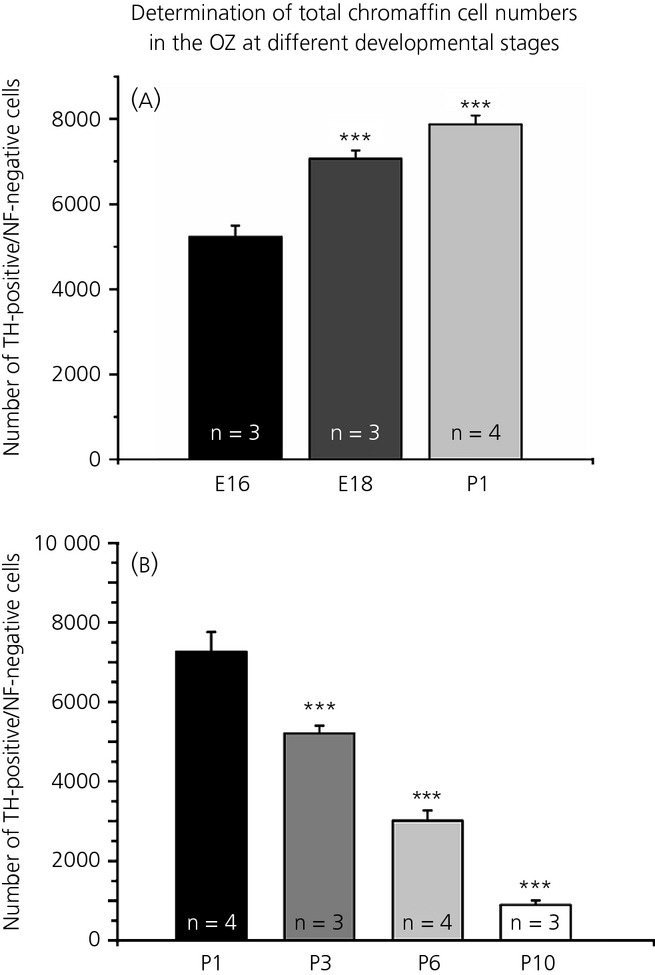
Evaluation of extra-adrenal chromaffin cell numbers located in the organ of Zuckerkandl (OZ) at pre-and postnatal developmental stages of wild-type mice. (a) Cell numbers increase from approximately 5000 at embryonic day (E) 16 to almost 8000 at postnatal day (P) 1. (b) Postnatally, cell numbers of the OZ decline to < 1000. ***P < 0.001.

We next analysed the pre- and postnatal development of the OZ again using combined NF-ISH and TH-IHC. As shown in Fig. 5(a–f), the OZ was most prominent during late embryonic ages, reaching its largest extension after birth/P1. Cell numbers increased from approximately 5000 (5231 ± 263) at E16 to almost 8000 (7872 ± 210) at P1 (Fig. 6a). The OZ subsequently decreased in size (Fig. 5d–f), becoming dissolved into small cell groups and single cells at P10, and cell numbers declined to less than 1000 (818 ± 67) at P10 (Fig. 6b). At P20, the OZ had virtually disappeared (not shown). Together, these data indicate a highly dynamic pattern in the pre- and postnatal development of the OZ.

### Lack of glucocorticoid receptor signalling accelerates postnatal loss of OZ chromaffin cells

Exogenously applied GC have consistently been reported to cause hyperplasia of extra-adrenal chromaffin tissue 27–29, although a role of endogenous GC for determining numbers of extra-adrenal chromaffin cells has not been shown. To address this issue, we investigated the impact of a deletion of the GR gene (*GR*^*DBHCre*^) on the postnatal development of the OZ.

**Fig. 7:**
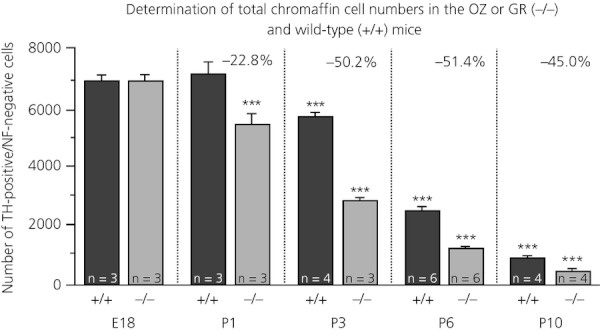
Evaluation of extra-adrenal chromaffin cell numbers located in the organ of Zuckerkandl (OZ) at embryonic day (E)18 and postnatal developmental stages in *GR*^*DBHC*^^*re*^ mice (−/−) compared to their wild-type littermates (+/+). Note acceleration in the postnatal numerical decline of chromaffin cells in the OZ. A significant linear cell loss in *GR*^*DBHC*^^*re*^ mice (−/−) has been documented, beginning from postnatal day (P) 3 onwards compared to the corresponding wild-type littermates (+/+). This observation indicates a physiological role of endogenous glucocorticoids (GC) for the postnatal maintenance of extra-adrenal chromaffin cells. (***P < 0.001).

Figure 7 reveals that numbers of chromaffin cells in wt and *GR*^*DBHCre*^ mice were still identical at E18 (wild-type: 6900 ± 185; GR (−/−): 6834 ± 243). Lack of GC signalling accelerates the postnatal numerical decline of chromaffin cells in the OZ. Cell losses in *GR*^*DBHCre*^ mice approximately double from P3 onwards compared to GR wild-types, suggesting a physiological role of endogenous GC for the postnatal maintenance of this extra-adrenal chromaffin cell population.

### Degeneration of chromaffin cells and autophagy

**Fig. 8:**
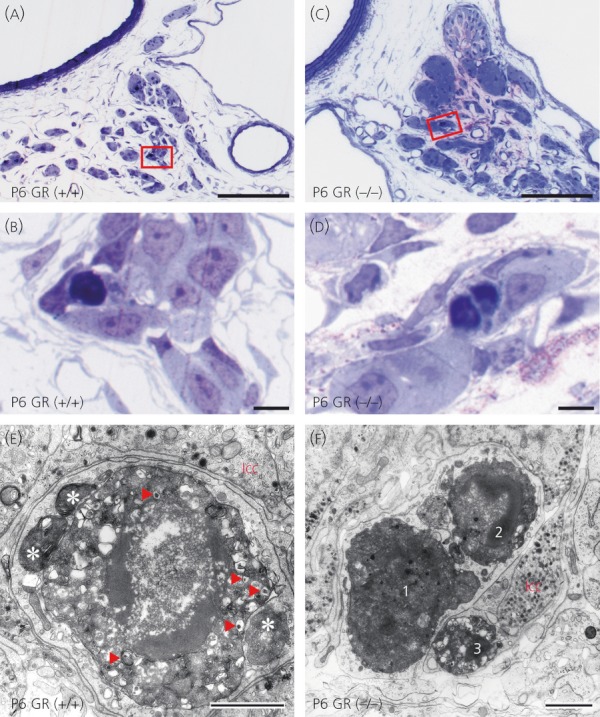
Light- and electron microscopic characterisation of degenerated chromaffin cells located in the organ of Zuckerkandl (OZ) of postnatal day (P) 6 *GR*^*DBHC*^^*re*^ mice (−/−) and age-matched wild-type littermates (+/+). (a–d) Toluidine blue stained semithin Epon sections of both, GC-receptor (GR) wild-type (a,b) and knockouts (c,d) reveal single cells and small cell clusters containing solid aggregates of dense material. Areas depicted by red frames (a, c) are enlarged in (b) and (d). Scale bars (a,c): 200 μm, (b, d): 10 μm. The same cells were identified by electron microscopy (e, f). (e) A degenerated chromaffin cell recognised by its typical chromaffin granules (red arrowheads) is engulfed by a narrow rim of cytoplasm from a cell that lacks chromaffin granules but contains three large lysosomes (stars). The nucleus of the chromaffin cell contains heterochromatin attached to the nuclear membrane. Icc, intact chromaffin cell. (f) Three degenerated chromaffin cells 1–3 contained within nonchromaffin cells. Icc, intact chromaffin cells. Scale bars (e, f): 5 μm (***P < 0.001).

**Fig. 9:**
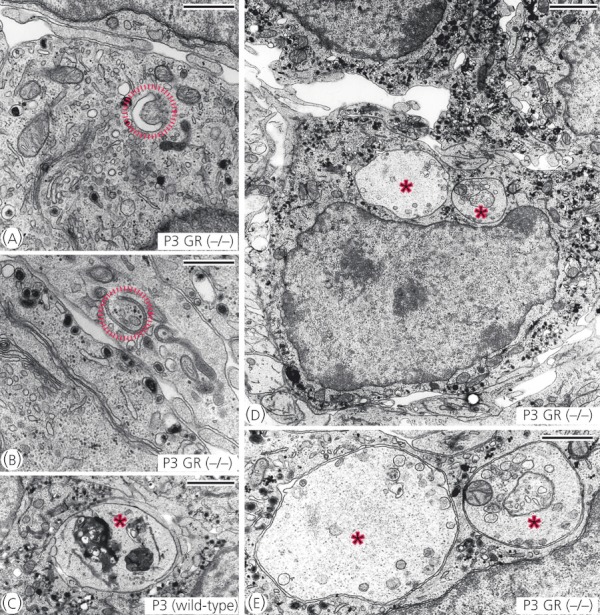
Electron microscopic characterisation of degenerated chromaffin cells located in the organ of Zuckerkandl (OZ) of postnatal day (P) 3 *GR*^*DBHC*^^*re*^ mice (−/−) and corresponding wild-type littermates (+/+). (a, b) Showing an initial stage of autophagy, the formation of the isolation membrane (red circles). (c–e) Showing advanced stages of autophagy (red stars); cytoplasmic inclusions (autophagosomes) containing dense bodies (c), cytoplasm, vesicles and mitochondria [(d), enlarged in (e)]. Scale bars (a, b, e): 1 μm, (c, d): 2 μm.

It has been suggested that postnatal regression of the OZ may be the result of dispersion by migration, loss of phenotype, ‘transdifferentiation’ into sympathetic neurones or cell death 7,14,15. We next analysed the cellular and subcellular structure of regressing OZs in both GR wild-type and knockout mice focusing on P6 and P3 by using serial semithin Epon sections. As shown in Fig. 8(a–d), Epon sections from both GR wild-type and knockouts (*GR*^*DBHCre*^) revealed single cells and small cell clusters containing solid clumps of dense material, corresponding to nuclei with condensed heterochromatin surrounded by remnants of cytoplasm with interspersed chromaffin granules (Fig. 8e,f). In most instances, these compacted corpses of chromaffin cells were surrounded by a rim of intact cytoplasm that lacked chromaffin granules, thereby tentatively defining these cells that had engulfed the chromaffin cell remnants as being ‘nonchromaffin’. Degenerated chromaffin cells amounted to approximately 10% of the total number of chromaffin cells at P6. At P3, we found a larger proportion of intact chromaffin cells (Fig. 9), many of which revealed ultrastructural signs of initial or advanced autophagy, such as autophagosomes (Fig. 9a,b), autophagolysosomes (Fig. 9c) and inclusion bodies within the cytoplasm containing cytoplasmic material, vesicles, and mitochondria (Fig. 9d,e).

### Detection of autophagy in tissue sections by IHC

**Fig. 10:**
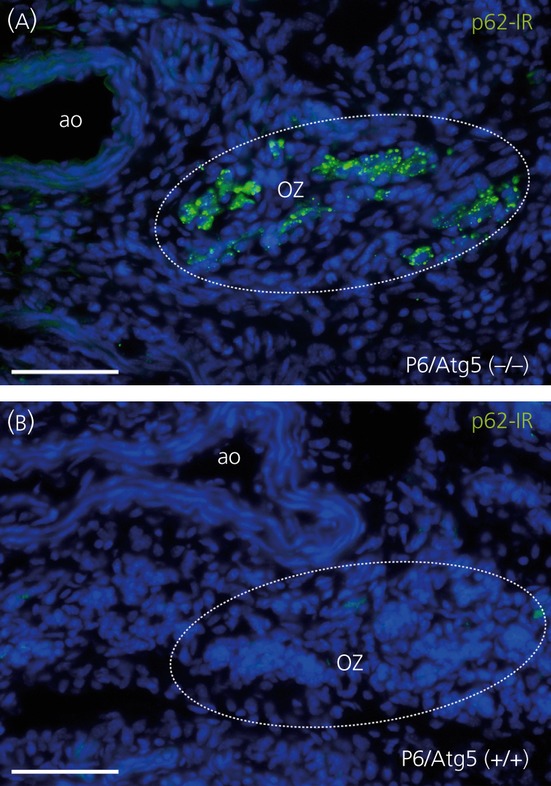
Immunohistochemical demonstration of accumulation of p62 protein in extra-adrenal chromaffin cells of organ of Zuckerkandl (OZ) (circled) in *Atg5*^*DBHCre*^ deficient mice (a). p62-immunoreactivity (-IR) accumulates, when autophagy is blocked, as in *Atg5*^*DBHC*^^*re*^ (−/−) mice. (b) p62-IR cannot be visualised in wild-type mice. Scale bars = 100 μm. ao, aorta.

Immunohistochemical markers known to be associated with autophagosome formation in cultured cells such as HEK cells (Fig. 1) [e.g. Atg5 42,43 and LC3 42,43] did not reveal positive signals in chromaffin cells of the OZ *in situ*. However, a pcAB to the p62 protein that operates as a selective substrate in autophagosome formation 44 and accumulates when autophagy is blocked (as in Atg5 deficient mice) 35,45 could be visualised in OZ extra-adrenal chromaffin cells of Atg5 mutant mice (*Atg5*^*DBHCre*^), as shown in Fig. 10. Thus, both electron microscopy and IHC data support the notion of ongoing autophagy in degenerating extra-adrenal chromaffin cells.

**Fig. 11:**
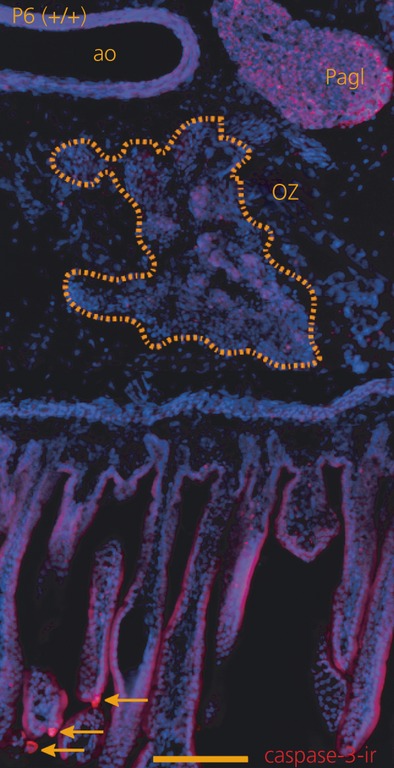
Immunohistochemical staining using an antibody against cleaved caspase-3 as a marker for apoptosis. The figure shows the lack of caspase-3-immunoreactivity (-IR) in the organ of Zuckerkandl (OZ) [postnatal day (P) 6 wild-type], contrary to caspase-3-IR in gut epithelial cells (yellow arrows). Scale bar = 200 μm. ao,aorta; pagl, paravertebral sympathetic ganglion.

To clarify the mode of death by which chromaffin cells die, we used terminal deoxynucleotidyl transferase dUTP nick end labeling (TUNEL) and staining for caspase-3, as well-established markers for apoptosis, without obtaining positive signals. Figure 11 shows the lack of caspase-3 IHC in the OZ, contrary to caspase-3 positive adjacent gut epithelial cells. It is worth noting that we also failed to detect the ultrastructural hallmarks of apoptosis (e.g. pyknosis) and nuclear fragmentation with normal morphological appearance of cytoplasmic organelles 46.

### Lysosomes and macrophages

**Fig. 12:**
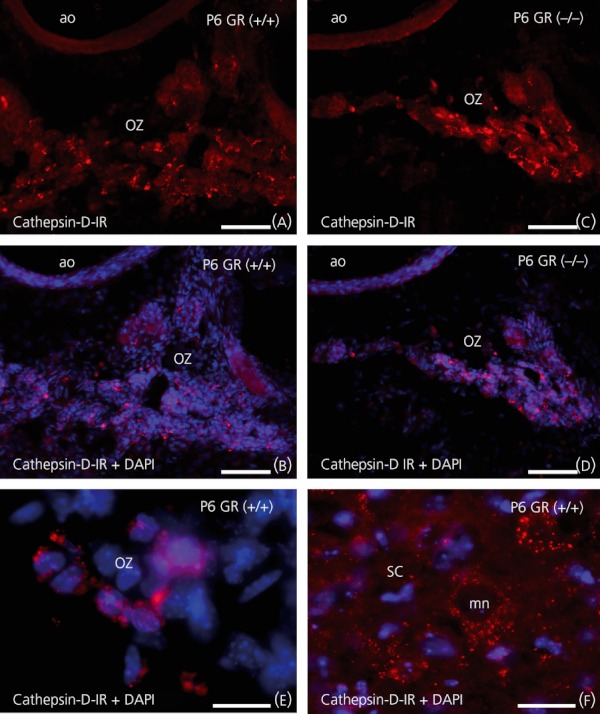
Immunohistochemical staining using an antibody to cathepsin-D as a marker for lysosomes. Note the more compact aggregates of immunoreactive material in the organ of Zuckerkandl (OZ) from *GR*^*DBHC*^^*re*^ compared to wild-type mice (a–d). The localisation of the compact immunoreactivity is unlikely to reflect lysosomes within chromaffin cells but rather cells surrounding chromaffin cells. A typical pattern of lysosome localisation in a neuronal cell (a spinal cord motoneurone) is depicted in (f). Together, these observations suggest that the aggregation of cathepsin-D-immunoreactivity could be related to invaded macrophages that surround chromaffin cells. Scale bars (a–d): 200 μm, (e, f): 20 μm. ao, aorta; mn, motoneuron; sc, spinal cord; DAPI, 4′,6-diamidino-2-phenylindole.

**Fig. 13:**
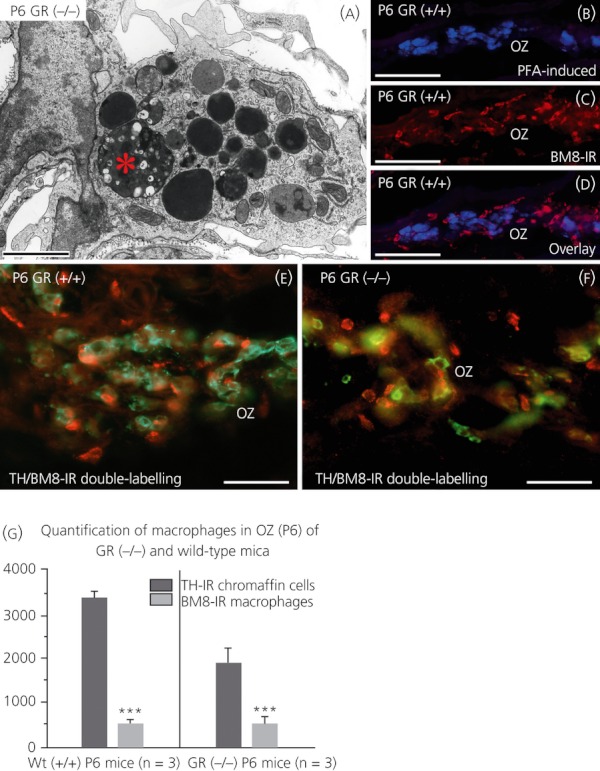
Light- and electron microscopic demonstration of macrophages inside the organ of Zuckerkandl (OZ) (a) shows the ultrastructure of a typical macrophage containing large lysosomes and a chromaffin cell fragment (red asterisk). (b–d) Showing cells that are strongly immunoreactive for the macrophage marker BM8. Chromaffin cells were identified by positive paraformaldehyde (PFA)-induced histofluorescence (b, d). (e, f) Double-labelling for tyrosine hydroxylase (TH)-immunoreactivity (-IR) and BM8-IR failed to reveal co-staining, indicating that both markers are located in distinct cell types and, furthermore, that chromaffin cells phagocytosed by macrophages are TH-IR negative. (g) Cell count data indicate that mice lacking the GC-receptor (GR) have almost identical numbers of macrophages in the OZ compared to wild-type (Wt) mice at postnatal day (P) 6. In the context of the decreased number of chromaffin cells in GR mutants (g), it indicates a higher density of macrophages inside the OZ. Scale bars (a): 5 μm, (b, c, d): 200 μm, (e, f): 100 μm. ***P < 0.001.

Staining for cathepsin-D-IR revealed more compact aggregates of immunoreactive material in the OZ from GR^DBHCre^ than wild-type mice (Fig. 12a,c). Cathepsin-D-IR was consistently located on the surface of chromaffin cells. Because lysosomes are usually evenly distributed within the cytoplasm of neurones and neuroendocrine cells and not concentrated peripherally, as confirmed in Fig. 12(f) for spinal cord motoneurones, we next investigated whether these accumulations of lysosomes in the OZ might be located within Schwann cells and/or macrophages surrounding chromaffin cells. Figure 13(a) shows the ultrastructure of a cell type frequently encountered within the postnatal OZ, which we tentatively identified as a macrophage containing large lysosomes and a fragment of a chromaffin cell (red asterisk). Figure 3(b,c) shows that cells strongly immunoreactive for the macrophage marker BM8 surrounded chromaffin cells identified by positive PFA-induced histofluorescence. Double-labelling for TH and BM8 failed to reveal co-staining, corroborating the notion that both markers are located in distinct cell types (i.e. chromaffin cells and macrophages) and, further, that chromaffin cells phagocytosed by macrophages are apparently devoid of detectable levels of TH-IR (Fig. 13e,f). Cell counts shown in Fig. 13(g) indicate that mice lacking the GR have almost identical numbers of macrophages in the OZ as compared to wild-type mice at P6. In the context of the decreased number of chromaffin cells in GR mutants, this indicates a higher density of macrophages, confirming the qualitative comparison seen in Fig. 12(a,c). Together, our data suggest that the faster regression of the OZ in GR mutants elicits enhanced activation of macrophages for efficient removal of autophagic chromaffin cells.

## Discussion

The OZ is the largest collection of extra-adrenal chromaffin cells 4. Its location apparently differs between species, most notably between humans 6 and rodents 4,12,13,18. In 1903, Kohn established that the OZ is of common origin with the chromaffin cells of the adrenal medulla. In terms of ultrastructure and chemical composition, chromaffin cells of the OZ are largely indistinguishable from adrenal chromaffin cells but lack phenylethanolamine N-methyltransferase and, consequently, adrenaline 3,17. In the human foetus, the OZ is much more prominent than the developing adrenal medulla. Secretion of catecholamines by chromaffin cells of the OZ and other paraganglia has been demonstrated 47–49, with vascular smooth muscle probably being the target and maintainenance of vascular tone a hypothetic function 7. Foetal extra-adrenal chromaffin cells appear to be stimulated directly by oxygen deficiency and not by preganglionic nerve input. This is consistent with the lack of synaptic nerve endings seen in the OZ. In infants, the OZ continues to increase in size until approximately 3 years and subsequently undergoes degenerative changes and atrophy.

It appears that the postnatal decrease in extra-adrenal chromaffin cell numbers correlates with plasma concentrations of GC. Plasma GC level peaks 2–3 days before birth in mice and is in a descending phase at birth 50; therefore, after birth, target tissues are exposed to less GCs. This level shows a significant rise by day 12, and then continues to rise, until peaking on day 24 in rats 51. We cannot exclude the possibility that the plasma GC levels are lower in *GR*^*DBHCre*^ mice mice after birth. However, the *GR*^*DBHCre*^ mice mice do not show different plasma levels of GCs in adulthood (R. Parlato, unpublished data) and we did not observe hypertrophy and hyperplasia of the cortical zones of the adrenal during early postnatal life 24. Rather, brain-specific GR mutants targeted also in the hypothalamic-pituitary axis die approximately 1 week after birth and display an increase in plasma corticosterone 52. During the stress hyporesponsive period occurring in mice after birth until day 12, GCs have low influence 53. The highly dynamic pattern in the pre- and postnatal development of the OZ suggests an association with the stress nonresponsive period; therefore, reduced extra-chromaffin cells in wild-type could depend on less GCs during this critical phase.

Postnatal involution of the OZ by ‘degeneration’, ‘dispersion’ or a ‘phenotypic conversion of chromaffin cells into sympathetic neurones’ has been described for all mammalian species investigated 4,12,13, although the cellular details and molecular bases of these processes have largely remained obscure. We now show that chromaffin cells in the OZ exhibit ultrastructural signs of autophagy. Autophagy is a cellular pathway serving the degradation of proteins and organelles and is primarily recognised as an important tool in a cell's pro-survival strategy 54–56. The process was first described in the 1960s as a bulk degradation system for removing proteins, lipids and organelles by the lysosomal pathway 57,58. The principal role of autophagy is to supply nutrients for cell survival and performance of quality control by inactivating misfolded proteins and nonfunctional organelles. Molecular genetic studies in the yeast *Saccharomyces cerevisiae* have helped to elucidate fundamentally important genes, the autophagy-related (Atg) genes that control autophagosome formation, a crucially important structure in autophagy 55. The distinct steps in autophagy can be followed by monitoring their ultrastructural features, which are key elements for the diagnosis of autophagy, and by molecular markers specific for the protein complexes that are recruited to these membrane structures 58. The first step in autophagy is the formation of a double-membrane structure, the isolation membrane (IM) or autophagophore, which expands and maturates into an autophagosome. The autophagosome fuses with lysosomes to form an autophagolysosome, which is subsequently degraded resulting in hydrolysation of the incorporated material. Our EM images of postnatal OZ chromaffin cells reveal an abundance of IM, autophagosomes and autophagolysosomes, which together suggest that degradation of chromaffin cells in the OZ is accompanied by autophagy.

We have failed to document immunoreactivities for Atg5 and LC3 *in vivo*. Even extensive modifications of the staining protocol, especially with regard to fixation conditions and the application of detergents in different concentrations, did not improve the outcome of our staining results. It should also be noted that the identification of autophagy-specific biomarkers by antibodies in tissues is highly compromised by low expression levels, which would require the use of an exogenous construct 59,60. In particular, the over-expression of GFP–LC3, in which GFP (green fluorescent protein) is expressed as a fusion protein at the amino terminus of LC3, has been succesfully applied in the past 61. Thus, LC3 AB can be used as a marker of autophagosome formation in tissue sections, although only when this protein is overexpressed 59. Similar constructs exist for Atg5 (YFP-Atg5, GFP-Atg5) 62. The *in vivo* expression level of Atg5 protein appears to be under physiological conditions similarly low and restricted to a short time window.

In addition to the established autophagy markers, it is also possible to use the protein p62/SQSTM1 as a marker for monitoring autophagy by IHC 35,45. The p62 protein serves as a link between LC3 and ubiquitinated substrates 63. p62 becomes incorporated into the completed autophagosome and is degraded in autolysosomes. Recent studies show that inhibition of autophagy correlates with increased levels of p62 protein, which can be visualised by specific antibodies 64,65 (Fig. 10). Using Atg5 deficient mice, in which autophagy is ablated and accumulation of p62 permitted, we were able to show that OZ extra-adrenal chromaffin cells undergo autophagy. Thus, two hallmarks of autophagy can be visualised in extra-adrenal chromaffin cells: first, the ultrastructural characteristics and, second, the accumulation of p62, once autophagy is blocked.

Macrophages, identified by ultrastructure, the macrophage marker BM8, and abundant immunoreactivity for cathepsin-D, a lysosomal marker, were abundant in the OZ engulfing chromaffin cells and fragments of chromaffin cells. The typical ultrastructure of chromaffin cells with their specific chromaffin granules remained recognisable even when the chromaffin cell is already largely fragmented and lacks TH-IR.

We were unable to detect signals documenting apoptosis in chromaffin cells of the OZ (e.g. TUNEL, activated caspase-3) and ultrastructural signs of apoptosis (e.g. nuclear pyknosis and fragmentation), together with normal morphological appearance of cytoplasmic organelles 46. Because visualisation of these apoptosis markers is limited to a relatively narrow time window, this may explain why apoptosis escaped our detection. However, an alternate possibility might be that autophagy itself marks a pathway towards cell death 46,66–69. The issue of autophagy as a pathway to cell death has not been satisfactorily resolved because cells undergoing different modes of death may activate an autophagy program for survival. However, the developmental death of neurones, such as the Rohon-Beard neurones and the lateral motoneurones in the lumbosacral spinal cord of the larval frog, and neurone death in the isthmo-optic nucleus of the chick embryo, have been described to occur with signs of ‘autophagic degeneration’ 70. It is also conceivable that OZ chromaffin cells may die under special modes of autophagy, such as necroptosis 71 or entosis 72. Both modes of cell death are accompanied by massive autophagy, although there are no reliable markers available to identify these types of death.

The present study clearly establishes that signalling through the GR is implicated in the maintenance of extra-adrenal chromaffin cells in the OZ. This is the first demonstration of a physiological role of GR signalling in the maintenance of extra-adrenal chromaffin cells, subsequent to the documentation of a role of GC in numerous ‘gain-of-function’ paradigms. Although our data add to the notion that GC signalling may be essential for the postnatal maintenance of both chromaffin cells within the adrenal gland and in paraganglia 24, it remains to be investigated whether this also applies to other chromaffin cell subpopulations (e.g. those in chemoreceptors).

Finally, mechanisms by which GC signalling activates a chromaffin cell survival programme are currently enigmatic, although they might be sought, for example, in signalling cascades linking the GR to the mitogen-activated protein kinase/extracellular signal regulated kinase pathway 73. GCs are known to provide neuroprotective effects on a variety of neurone populations. One mechanism by which this occurs involves activation of trk neurotrophin receptors 73. Chromaffin cells express the nerve growth factor (NGF) receptor trkA 31. NGF and dexamethasone share the capacity to promote survival of chromaffin cells *in vitro* 1. Thus, NGF may be a candidate that mediates protective effects of GC on chromaffin cells. Activation of the GR has also been shown to induce expression of the pro-survival protein IAP2 74. Irrespective of the mechanisms underlying the protective effects of GC, the resulting survival programmes antagonise two apparently different death scenarios: apoptotic death without autophagy in the GR-deprived adrenal gland 24 and death with autophagy in the extra-adrenal chromaffin cells of the OZ.
